# Defensive Metabolites from Antarctic Invertebrates: Does Energetic Content Interfere with Feeding Repellence?

**DOI:** 10.3390/md12063770

**Published:** 2014-06-24

**Authors:** Laura Núñez-Pons, Conxita Avila

**Affiliations:** Department of Animal Biology (Invertebrates) & Biodiversity Research Institute (IrBio), Faculty of Biology, University of Barcelona, Av. Diagonal 643, Barcelona ES-08028, Catalonia, Spain; E-Mail: conxita.avila@ub.edu

**Keywords:** chemical ecology, marine natural products, amphipod *Cheirimedon femoratus*, hexactinellid sponges, colonial ascidians, soft corals, chemical defense

## Abstract

Many bioactive products from benthic invertebrates mediating ecological interactions have proved to reduce predation, but their mechanisms of action, and their molecular identities, are usually unknown. It was suggested, yet scarcely investigated, that nutritional quality interferes with defensive metabolites. This means that antifeedants would be less effective when combined with energetically rich prey, and that higher amounts of defensive compounds would be needed for predator avoidance. We evaluated the effects of five types of repellents obtained from Antarctic invertebrates, in combination with diets of different energetic values. The compounds came from soft corals, ascidians and hexactinellid sponges; they included wax esters, alkaloids, a meroterpenoid, a steroid, and the recently described organic acid, glassponsine. Feeding repellency was tested through preference assays by preparing diets (alginate pearls) combining different energetic content and inorganic material. Experimental diets contained various concentrations of each repellent product, and were offered along with control compound-free pearls, to the Antarctic omnivore amphipod *Cheirimedon femoratus*. Meridianin alkaloids were the most active repellents, and wax esters were the least active when combined with foods of distinct energetic content. Our data show that levels of repellency vary for each compound, and that they perform differently when mixed with distinct assay foods. The natural products that interacted the most with energetic content were those occurring in nature at higher concentrations. The bioactivity of the remaining metabolites tested was found to depend on a threshold concentration, enough to elicit feeding repellence, independently from nutritional quality.

## 1. Introduction

One main topic in the field of marine chemical ecology is chemical defense, especially the investigation of secondary metabolites that provide protection from predation in potential prey. A series of questions arise when studying feeding repellents: how do they affect predators, are they toxic, or do they taste bad? Do chemicals require characteristic moieties to interact with specific receptors to be active as defenses? Are certain types of molecules more effective than others? What range of predators do metabolites affect? Are repellents energetically costly? May a secondary metabolite have no function? Do other metabolites (e.g., co-occurring bioactive secondary or primary metabolites, nutrients) interact with feeding repellents altering (enhancing or reducing) their effects? [1,2,3]. Some of these questions were approached in the present study under laboratory controlled conditions, using different isolated forms (pure compounds or mixtures of related metabolites) of chemical defenses, and assessing their levels of bioactivity and interaction with nutrients.

Predation is a dominant force in controlling populations of marine invertebrates, and prey organisms have evolved protective strategies, ranging from behavioral (nocturnal activity, rapid escape) and physical (spines, armor), to chemical mechanisms [4]. It is believed (but not fully proved) that palatability/distastefulness, and not toxicity, is responsible for the action of most defensive repellents, which elicit immediate aversive responses in potential predators [5,6,7]. The assumption of a metabolic cost for possessing repellents is ambiguous. Very few studies have examined the metabolic investments of defense in relation to an organism’s energy budget (for maintenance, growth, reproduction). Energy may be saved if defenses are active in small concentrations, do not require detoxification for storage, or if they originate from dietary or symbiotic producers. To optimize costs, chemical defenses could be differentially allocated in parts that are more susceptible to attacks, or be produced only when needed, as predicted by the optimal defense theory (ODT) [8]. There are also secondary metabolites that accumulate as by-products of the synthesis of other compounds, and have no known specific function [9]. Indeed, from the enormous diversity of chemicals from benthic invertebrates, ecological function has only been established in a tiny fraction of them [2,6,10,11]. 

The activity of antipredatory compounds might be altered by the presence of other compounds or physical devices, sometimes acting in an additive or synergistic manner. However, evaluating such effects is of great complexity, as is illustrated in an exchange of controversial publications [12,13,14]. Dietary components, like attractants that enhance feeding (*i.e.*, amino acids, nutrients), may instead reduce repellent activity, interacting in an antagonistic way. The nutritional quality of potential prey is therefore relevant, since it is likely that the same sensory processes that predators use to reject defensive metabolites are also used to perceive nutrients. Thus, prey with low nutritional quality may be rejected at lower levels of chemical defense, and conversely, natural products might only be deterrent at high concentrations in more nutritious items. Experimentally, high energy assay foods may mask minimally effective defensive metabolites, and consequently, products with weak bioactive properties may only cause deterrence along with low energy foods [15,16]. 

Furthermore, the relationships between chemical structure and defensive activity have not yet been addressed. Compounds of several classes and different polarities are described among feeding deterrents, from non-polar terpenoids to polar glycosides, some with broad effectiveness against a wide range of predators [2,6,10,17,18,19]. This suggests that chemoreceptive responses of diverse predatory taxa are similar at the molecular level [7]. There are likewise metabolites displaying multiple defensive roles, for example antipredatory and antifouling [20,21], representing energy saving defenses against different potential enemies, according to the Optimality Theory (OT) [22].

For all intents and purposes, most of the questions listed above remain unsolved, and, so far, little information is available. Besides, the research effort to understand antipredatory defensive strategies is notably biased, since it has been much more intensive in zones like the Caribbean than in other marine regions, in particular the Poles [1,2,3,6]. Research on the Southern Ocean regarding ecological interactions mediated by chemistry has grown in the past years, and many natural products have been discovered, some with allelochemical functions (e.g., for reviews see [10,11,17,18,23]). The present study attempts to elucidate some of the obscure points yet to be understood, on how feeding repellents operate in the presence of distinct relative amounts of nutrients, focusing on a benthic predator–prey scenario in a remote geographic area, Antarctica. In these polar ecosystems, predation (driven by invertebrate consumers, such as sea stars, nemertine worms and dense amphipod populations) is a selective force structuring benthic communities [24]. Following our previous investigations on defensive metabolites from Antarctic invertebrates with feeding repellent properties, the aims of this work are: (1) to evaluate how the antipredatory activity of selected natural products is affected by the energetic content of food (usually expressed in units of energy per mass; e.g., kJ·g^−1^); (2) to compare the potency/efficacy of the compounds tested as defenses, according to their chemical structure; and (3) to determine whether the efficiency of defensive metabolites (compound concentration required to yield bioactivity) has any relationship with the concentrations in which they appear in nature, and/or interference with the nutritional value of assay food items designed in order to mimic possible prey. The target compounds included alkaloids (meridianins A–G) from *Aplidium* ascidians [21,25], a steroid (5α(*H*)-cholestan-3-one) from hexactinellid sponges [26], wax esters from *Alcyonium* soft corals [27], a meroterpenoid (Rossinone B) from the ascidian *A. fuegiense* [21,28], and a new organic acid, glassponsine, from the glass sponge *Anoxycalyx* (*Scolymastra*) *joubini* [29]. These products were included in artificial diets of different energetic values, at distinct concentrations, and were then presented to a sympatric Antarctic amphipod predator in feeding experiments. Due to its ecological relevance as generalist consumer, we chose the lyssianasid *Cheirimedon femoratus* as the experimental predator for our assays.

## 2. Results and Discussion

**Figure 1:**
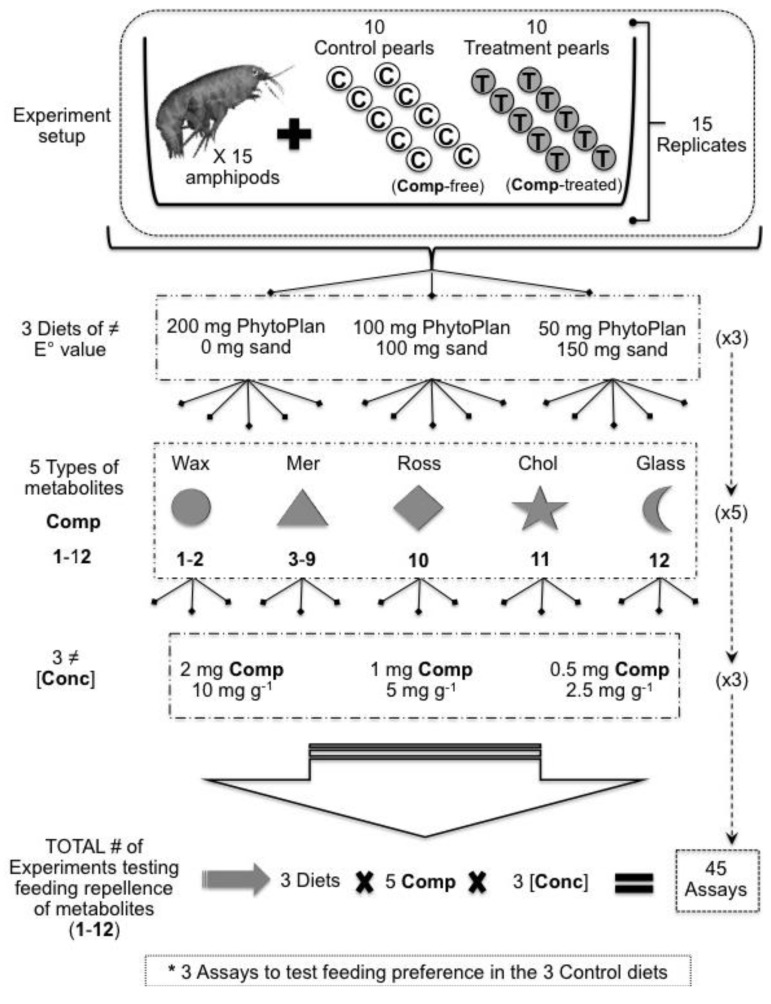
Diagram of the experimental design. In total, 45 feeding preference experiments were performed with the amphipod *Cheirimedon femoratus* to test the repellent activities of five types of metabolites, **Comp** (**1**–**12**). These were incorporated in three assay alginate-based food pearls of distinct energetic values, at three different testing concentrations (3 diets × 5 **Comp** × 3 [**Conc**] = 45 bioassays). The five compound types are symbolized with icons: “Wax”—circle: Wax esters (**1**–**2**); “Mer”—triangle: meridianins A–G (**3**–**9**); “Ross”—rombe: rossinone B (**10**); “Chol”—star: 5α(*H*)-cholestan-3-one (**11**) and “Glass”—moon: glassponsine (**12**). * In addition, three experiments assessed the feeding preference among the three control (compound-free) assay diets.

Sufficient material (≈11 mg) of each of the isolated target compounds (**10**–**12**), and of the target mixtures of chemically related products (**3**–**9**) and (**1**–**2**), was successfully obtained for the experiments. Both mixtures are composed of metabolites of the same family type: Within the wax fraction, both wax esters **1** and **2** appear in approximately the same proportion ~1:1 [27]; whereas the meridianin (A–G) alkaloid mix (**3**–**9**) contains a major component constituted by meridianins A–E (**3**–**7**) and a minor one corresponding to F–G meridianins (**8**–**9**), in a relative proportion of ~94:6. These proportions may exhibit slight variabilities among colonies. Nevertheless, the isolated forms of A–G meridianins have demonstrated in the past similar efficiency as feeding repellents [25]. The natural relative concentrations of the metabolites within both mixtures (**1**–**2** and **3**–**9**) were maintained (not modified after fractionation) in the bioassays here performed. The natural products **1**–**11** had been previously isolated from our Antarctic sample collections, all of them demonstrating feeding repellent properties towards relevant sympatric predators: the sea star *Odontaster validus* and the amphipod *Cheirimedon femoratus* [21,25,26,27]. In preliminary experiments, the newly described glassponsine (**12**) did not cause rejection in feeding tests at its natural concentration (~2.34 mg·g^−1^; [29] and present study), but repellent activity was confirmed at higher concentrations (see below). Specific data of the five target compound types can be found in Table 1, while the information on the setup design of the experiments is illustrated in Figure 1. 

As a consequence of exhaustive analyses, glassponsine (**12**) was recently recovered as a new compound from several individuals of the common Antarctic Hexactinellid sponge *Anoxycalyx* (*Scolymastra*) *joubini* [29], and was here assessed as a feeding deterrent product for the first time. Conspecific samples from collections coming from different geographic areas have never afforded molecules similar to **12** [10,26]. Glassponsine **12** was recovered as an abundant sulfonate salt (see Table 1). It has a close structural relationship with the widely distributed taurine, responsible for many biological roles in animals (*i.e.*, osmolytes in deep-sea organisms) and a major constituent of bile [30]. Related sulfonate acids, some found in demosponges and also common in bacterial membranes, are important antibiotics in the production of sulfa drugs [10,11,31]. The scant organic content and high spicule concentration of glass sponges make them supposedly already discouraging to predators, and needless to produce secondary metabolites [32]. Frequently though, marine organisms reveal variability in chemical profiles and in defensive activities within the species or even population level, and this is often attributed to symbiotic producers [33,34,35,36]. Bacterial symbionts, due to the extremely reduced mesohyl of Hexactinellida, are believed to be unsubstantial in this class of Porifera [32] when compared with Demospongiae [37]. Glassponsine might originate from microbial associates, or either be produced by host cells of a distinctive population of glass sponges adapted to particular conditions, and from which our specimens were recovered. In any case, from an ecological evolutionary perspective, intraspecific variabilities in bioactivity patterns may promote segregation, favoring the selection for certain defended phenotypes. These issues still require further investigation. 

Analysis with Thin Layer Chromatography (TLC) were used to evaluate chemical changes in the alginate pearls during experimentation; these showed that the metabolites added to the artificial feeding pearls mixtures always remained unchanged after the bioassays. 

**Table 1:** Chemical and biological data of the five types of metabolites tested (**1**–**12**). [N]_Comp_ DW_TOTAL_: Compound natural concentration ([N]_Comp_) with respect to sample’s total dry weight (DW_TOTAL_= DW + EE + EB; where DW: residual dry weight, EE: ether extract, BE: butanol extract).

Compounds’ Name and Chemical Structure	Data	
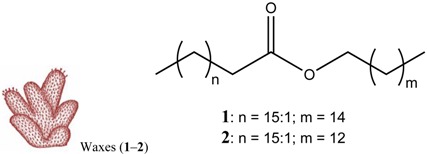	Chemical type	Wax ester
	[N]_Comp_ in DW_TOTAL_	25 mg·g^−1^
	Organic fraction	Ether (EE)
	Source organism	Soft coral
	Species name	*Alcyonium haddoni*
	Phylum:Class	Cnidaria:Anthozoa
	Sample location	Deception Island
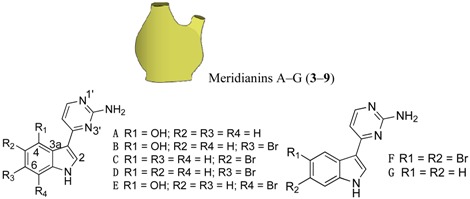	Chemical type	Indole alkaloid
	[N]_Comp_ DW_TOTAL_	24.3 mg·g^−1^
	Organic fraction	Ether (EE)
	Source organism	Colonial ascidian
	Species name	*Aplidium falklandicum*
	Phylum:Class	Chordata:Ascidiacea
	Sample location	Weddell Sea
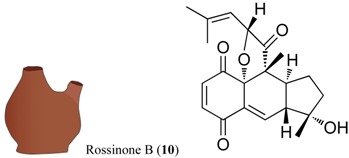	Chemical type	Meroterpene
	[N]_Comp_ DW_TOTAL_	5.1 mg·g^−1^ *
	Organic fraction	Ether (EE)
	Source organism	Colonial ascidian
	Species name	*Aplidium fuegiense*
	Phylum:Class	Chordata:Ascidiacea
	Sample location	Weddell Sea
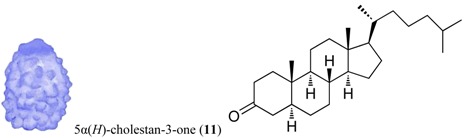	Chemical type	Keto-steroid
	[N]_Comp_ DW_TOTAL_	2.8 mg·g^−1^
	Organic fraction	Ether (EE)
	Source organism	Glass sponge
	Species name	*Rossella nuda*
	Phylum:Class	Porifera:Hexactinellida
	Sample location	Weddell Sea
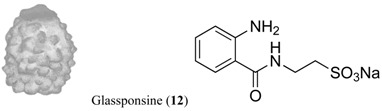	Chemical type	Sulfonate acid
	[N]_Comp_ DW_TOTAL_	2.34 mg·g^−1^
	Organic fraction	Butanol (BE)
	Source organism	Glass sponge
	Species name	*Anoxycalyx* (*Scolimastra*) *joubini*
	Phylum:Class	Porifera:Hexactinellida
	Sample location	Weddell Sea

[N]_Comp_ values refer to average yields obtained from this study and previous publications [21,25,26,27]; * Natural concentrations of rossinone (**10**) come from inner regions of the colonies after dissection.

### 2.1. Control (Compound-Free) Diets

**Figure 2:**
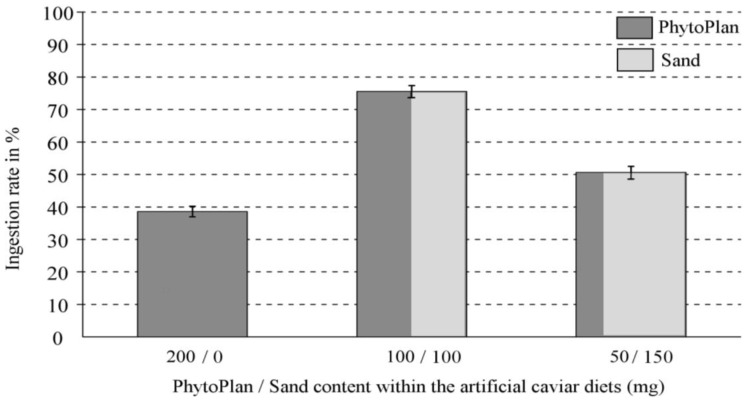
Average ingestion rates and corresponding standard errors in percent values (%). This data compiles the consumption by the Antarctic amphipod *Cheirimedon femoratus* during the 45 feeding preference tests (5 h food exposure) on the three control (compound-free) assay diets, based on alginate pearls. The different prepared foods correspond to pearls containing 200, 100 and 50 mg of PhytoPlan^®^ feeding stimulant each, along with a compensating quantity of sand to maintain total dry weight values, which according to the diets were 0, 100 and 150 mg, respectively. Ingestion rates were all significantly different according to one-way ANOVA and *post hoc* Tukey (HDS) (*p* ≤ 0.001 **).

The three prepared alginate-based food pearls, containing different proportions of feeding stimulant (Phytoplan^®^) and sand, produced significantly different ingestion rates in the amphipod *Cheirimedon femoratus* (Figure 2) according to the one-way ANOVA analysis (*F*_(3,2)_ = 38.107, *p* < 0.001 **). The energetically intermediate diet (100 mg feeding stimulant, 100 mg sand) was the most consumed (*p* < 0.001 ** Tukey (HDS) *post hoc* tests, when compared with the other two diets). The least nutritive food pearls (50 mg feeding stimulant, 150 mg sand), were more intensively eaten than those with 200 mg Phytoplan^®^ and no sand added (*p* < 0.001 **), thus ranking ingestion rates per diet are: 100 > 50 > 200 mg Phytoplan^®^. When testing preferences between the three diets, the energetically richest pearls (200 mg Phytoplan^®^) were significantly preferred compared with the other two feeding pearls (*p* < 0.05 * in both comparisons according to Wilcoxon Exact tests). Pearls prepared with 100 mg food source tended to be preferred compared with those of 50 mg Phytoplan^®^ and 150 mg sand, yet the difference was not significant (*p* = 0.089). These preferences may be resumed as: 200 > 100 ≥ 50 mg Phytoplan^®^ (see Table 2; Figure 2). Consequently, amphipods prefer the energetically richest (200 mg Phytoplan^®^) diet, but require lesser amounts to reach saturation compared to the lower energy diets.

**Table 2:** Comparative results of ingestion and preference for the three control assay diets (compound-free) of different energetic content offered to the Antarctic amphipod *Cheirimedon femoratus*. Differences in ingestion rates were calculated applying a one-way ANOVA analysis, followed by *post hoc* Tukey (HDS); and differences in feeding preferences using the Wilcoxon Exact Test.

Diets Contrasted	Feeding Preferences		Ingestion Rates	
PhytoPlan^®^ Content	Preference Result	Wilcoxon Test	Ingestion Result	Tukey (HDS)
200 mg *vs.* 100 mg	200 mg > 100 mg	*p* = 0.036 *	200 mg < 100 mg	*p* < 0.001 **
200 mg *vs.* 50 mg	200 mg > 50 mg	*p* = 0.014 *	200 mg < 50 mg	*p* < 0.001 **
100 mg *vs.* 50 mg	100 mg > 50 mg	*p* = 0.089 n.s.	100 mg > 50 mg	*p* < 0.001 **

Feeding preferences of the amphipod *Cheirimedon femoratus* were towards the energetically richest non-mineralized diets, in comparison with poorer foods containing inorganic material reflected palatability (feeding attractiveness); whereas the pattern of ingestion, with highest consumption of energetically intermediate foods, and lowest with most nutritive ones, is likely related to satiation and digestibility. While nutritious items are probably more attractive, their high energetic values make them also more satiating, leading to a lower consumption when compared with less nutritive diets. Alternatively, energetically poor food items, with high inorganic content, may cause lower ingestion rates than what would be expected, due to an excess of indigestible inert load, making their digestion too costly, less profitable, and therefore not worthwhile for consumers. Compensatory feeding is a well-described phenomenon by which animals compensate for ingesting energetically poor diets by increasing the rate of consumption. Indigestible components (sand) in low nutrient density foods might, however, offset compensatory ingestion, since an enhanced intake also entails accumulation of profitless substances [38,39]. Simultaneously, according to the digestive rate model (DRM), predators select for most nutritive, easily digestible items [7,40,41]. Our findings are in agreement with those predictions; food with intermediate energetic and inorganic content provided the highest ingestion rates and intermediate feeding preferences. 

### 2.2. Feeding Repellent Activities of Target Bioactive Metabolites (**1**–**12**)

Activities of the five different metabolite types towards the amphipod *Cheirimedon femoratus* differed depending on concentration and diet energy content (Table 3; Supplementary Figure S1). The meridianin A–G (**3**–**9**) mixture was the most active (Wilcoxon Exact Tests; *p* < 0.05 *), causing repellency to the amphipod in seven of the nine assays performed (77.8% active tests). No deterrence was observed, only in tests with the intermediate and lowest compound concentrations (5, 2.5 mg·g^−1^) and added to the richest diet (pearls made with 200 mg Phytoplan^®^ and no sand). Glassponsine (**12**) with 33.3%, active results, and rossinone B (**10**) with 44.4% were repellent at their highest concentrations (10 mg·g^−1^) in all three types of artificial foods. Rossinone B (**10**) was also repellent when incorporated to pearls of highest energetic values (200 mg Phytoplan^®^) at intermediate concentration (5 mg·g^−1^). The steroid 5α(*H*)-cholestan-3-one (**11**) yielded 11.1% incidence of activity, only eliciting repellence at the highest compound concentration (10 mg·g^−1^) included in the energetically richest diet type (200 mg Phytoplan^®^). Finally, the wax esters (**1**–**2**) were similarly active only in one test (11.1% activty), but only when incorporated at highest experimental concentration (10 mg·g^−1^) within pearls of lowest nutritional content (50 mg Phytoplan^®^ and 150 mg sand). These results are summarized in Table 3 and Supplementary Figure S1. 

**Table 3:** Results of the 45 feeding preference assays performed with the target compounds (**1**–**12**) and using the Antarctic amphipod *Cheirimedon femoratus* as model predator. The experiments tested repellency of each of the five types of metabolites at three different concentrations, included in assay food pearls of three different energetic values. Feeding repellent activities were calculated according to significant differences in consumption rates between paired control compound-free and compound containing treatment diets, analyzed with Wilcoxon Exact Tests. Colored boxes with “+” sign: Active in feeding repellence (*p* < 0.05 *); white boxes with “−” sign: Inactive (*p* ≥ 0.05 n.s.).

Compounds			(mg·g^−1^)		PhytoPlan (mg)					
					200		100		50	
Wax esters (**1**–**2**)			10		**−**		**−**		**+**	
			5		**−**		**−**		**−**	
			2.5		**−**		**−**		**−**	
Meridianins A–G (**3**–**9**)			10		**+**		**+**		**+**	
			5		**−**		**+**		**+**	
			2.5		**−**		**+**		**+**	
Rossinone B (**10**)			10		**+**		**+**		**+**	
			5		**+**		**−**		**−**	
			2.5		**−**		**−**		**−**	
5α(*H*)-cholestan-3-one (**11**)			10		**+**		**−**		**−**	
			5		**−**		**−**		**−**	
			2.5		**−**		**−**		**−**	
Glassponsine (**12**)			10		**+**		**+**		**+**	
			5		**−**		**−**		**−**	
			2.5		**−**		**−**		**−**	
	**0%–20%**	**21%–40%**		**41%–60%**		**61%–80%**		**81%–100%**		

* Color codes corresponding to percentage intervals of relative average differences of ingestion rates between control *vs.* treatment food pearls for the 15 replicate tests of each experiment.

### 2.3. Interference between Energetic Content and Compounds (**1**–**12**) Deterrent Bioactivity

**Figure 3:**
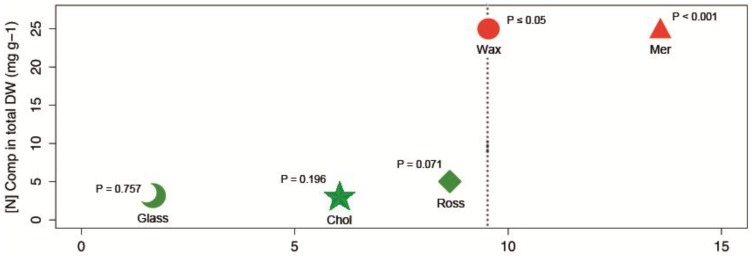
Relationship between the natural concentration in total dry weight ([N] **Comp** in total DW) *vs.* the likelihood ratio statistic G calculated in G-Tests, for the five compoundstypes (**1**–**12**) analyzed. The G value, from 3 × 3 contingency tables, provides the grade of dependence/interaction between the three assay diets and compound experimental concentrations ([**Conc**]’s) in relation to the repellent bioactivities recorded for the target metabolites and the interference with food energetic content. The five compound types are symbolized with icons: “Wax”—circle: Wax esters (**1**–**2**); “Mer”—triangle: meridianins A–G (**3**–**9**); “Ross”—rombe: rossinone B (**10**); “Chol”—star: 5α(*H*)-cholestan-3-one (**11**) and “Glass”—moon: glassponsine (**12**). *p*-Values are provided, and compounds’ icons are represented in red when the G-Tests yielded significant interference between energetic content and bioactive metabolite concentrations (*p* < 0.05 *), or in green when there was no statistical significance for interaction in the tests (*p* > 0.05 n.s.). The dotted line marks the limit of statistical significance along the Y-axis (G value).

According to G-Tests of independence calculated on 3 × 3 contingency tables constructed with the variables “Assay diet” and “Compound experimental concentration”, there were different levels of interaction between the artificial feeding pearls’ energetic content and compound efficacy as repellents for the five compound types assessed (**1**–**12**). Meridianins **3**–**9** exhibited the highest grade of interference in their feeding repellent properties (G = 13.5546; *p* < 0.01 **, Figure 3), experiencing a reduction in the efficiency (e.g., requirement of higher compound concentrations to provide deterrence) when the energetic content of assay diets increased. The next product whereby bioactivity was offset by energy content was the wax esters **1**–**2** (G = 9.493; *p* < 0.05 *). For the rest of the metabolites analyzed, Rossinone **10**, Cholestan **11**, Glassponsine **12**, no statistic significance (G = 8.626, 6.037, 1.884 respectively; *p* > 0.05 n.s.) supported the existence of dependence/interaction between the experimental diets and bioactive compound concentrations; e.g., reduced efficacy of defensive metabolites as antifeedants in higher energy assay food items (Figure 3). 

* The larger the likelihood ratio statistic G is, the larger is the grade of interaction between variables (*i.e.*, “Assay diets” *vs.* “Compound testing concentrations”), and the lower is the *p*-value.

Glassponsine (**12**) showed no feeding repellent activity at 2.5 mg·g^−1^, close to its natural concentration (2.34 mg·g^−1^), but highest assay concentrations (10 mg·g^−1^) did elicit rejection by *Cheirimedon femoratus*. The ketosteroid 5α(*H*)-cholestan-3-one (**11**) is another compound from Antarctic glass sponges, in this case *Rossella nuda*, and it appears in variable amounts in several species of hexactinellids. This metabolite was reported as an effective repellent at a mean concentration of 2.5 mg·g^−1^, yet its defensive role is considered to be minor and likely supported by synergism with other co-occurring agents [26]. It shows unpredictable levels of activity, since no correlation is found between concentration and feeding rejection in the sponge extracts containing the steroid [26]. 5α(*H*)-cholestan-3-one, like other steroid ketones, is a by-product of the cholesterol degradation route, and its accumulation is a matter of debate, as either a metabolic dead end or as a discrete functional defense [9,42]. In the present study, only one experiment yielded significant unpalatability at the highest compound concentration. 

Wax esters (**1**–**2**) were purified from *Alcyonium haddoni*, but they are frequent in Antarctic *Alcyonium* soft corals in general [27], as well as in other marine organisms. Wax esters are the main energy resources in anthozoans (corals), and are significantly dependent on the metabolic demands of the colony, which explains their highly variable tissue concentrations [43]. They are indigestible for most consumers, and possess additional unpalatable activity towards relevant Antarctic predators at diverse tissue concentrations, starting at ~5 mg·g^−1^ for *C. femoratus* amphipods, according to previous studies [27]. In the experiments here performed, however, only in one case did high wax concentration (10 mg·g^−1^) provoke amphipods to reject feeding pearls (Table 1 and Table 3; Supplementary Figure S1). Wax esters **1**–**2** play a major primary metabolic function as lipidic reserves, while providing deterrent properties; this dual role turns them into efficacious energy-saving defensive metabolites. Similarly to the aforementioned ketosteroid **11**, waxes are thought to cooperate in an additive way with other more potent bioactive secondary metabolites. In corals, they presumably interact with terpenoids (e.g., illudalane alcyopterosins in Antarctic *Alcyonium*), to provide a more effective protection [27]. 

The remaining products consisted of potent chemical defenses coming from ascidians and of secondary metabolism origin. Rossinone B (**10**) was first described from an Antarctic colonial *Aplidium sp.* [44], and was subsequently isolated from *Aplidium fuegiense* [28]. It is a meroterpenoid preferentially stored towards the internal parts of the colonies, and with strong feeding repellent properties at inner natural concentrations (5.1 mg·g^−1^) [21,28]. In the present study, rossinone B displayed activity with the highest and intermediate concentrations (5 and 10 mg·g^−1^), but not in all cases for the intermediate amount. Finally, meridianins A–G (**3**–**9**) are indole alkaloids originally reported from *Aplidium meridianum* [45], and later discovered in *Aplidium falklandicum* [25]. Meridianins are very potent protein kinase inhibitors of pharmacological interest [11,46]. Ecologically, they have multiple defensive roles that extend beyond antipredatory to antifouling, presumably turning them into multipurpose repellents [22]; however, their high tissue amount puts into question the energy-saving theory, unless they are produced by symbionts. In nature, meridianins occur as a complex mixture of related alkaloids, separately exhibiting some types of bioactivity at concentrations as low as 0.75 mg·g^−1^ [25]. Several other repellents that are effective as isolated forms, (e.g., tambjamines, water-borne steroids) appear normally as families of related minor metabolites, and have enhanced effects in combination. Apparently, producing mixtures of similar active chemicals adds more signals to the bioactive constituent [25,47,48]. Meridianins A–G occurred in abundant quantities here, up to 24.3 mg·g^−1^ distributed throughout the whole colony (in accordance to previous studies [21,25]), yet frequently they may be slightly more concentrated towards outer regions. They are impressively powerful feeding repellents, even at substantially lower concentrations than those calculated as natural [21,25]. In this study, the meridianin mixture was the most effective repellent causing amphipod rejection at all testing concentrations (see Table 1 and Table 3; and Supplementary Figure S1). 

The evolution of chemical defenses and the responses exhibited by consumers cannot be understood in isolation from dietary ingestion. Similarly, nutrition must integrate the effects of non-nutrient components, including indigestible substances and secondary metabolites. One mode of antipredatory defensive strategy is proposed to use this antagonistic interaction between nutrients and repellents. Thus, prey may become able to reduce palatability by combining low nutrient content with the presence of distasteful secondary metabolites. Following these assumptions, chemical defenses should be more (or only) effective when incorporated into nutritionally low-quality diets. However, there is still scant empirical evidence, with only a few compounds tested, to support these arguments [7,12,16]. Our experimental data show that levels of repellent activity vary for each compound type (**1**–**12**), and that they perform differently when mixed with nutritionally distinct assay foods. The products that interacted the most with energetic content were those that occur in nature at higher tissue concentrations, according to G-Tests of independence (Figure 3). Those were the wax esters (**1**–**2**) and meridianins A–G (**3**–**9**). More energetic diets required higher quantities of these metabolites to produce repellent effects; efficient bioactivity, however, was acquired with meridianins at much lower concentrations when compared with wax esters. Accordingly, waxes were only active at maximum concentrations in the poorest diets; whereas meridianins always elicited repellency, except with the most nutritious diets, for which maximum concentration of the mix was needed to provide effective amphipod rejection. Instead, rossinone B (**10**), 5α(*H*)-cholestan-3-one (**11**) and glassponsine (**12**), which appear in much lower quantities (five- to ten-fold lower) within the source organisms, behaved independently with respect to food quality (see Table 3 and Figure 3). For these compounds maximum concentrations were active irrespective of the diet richness, except for the ketosteroid (**11**), which was surprisingly only active in most nutritious pearls. Rossinone B (**10**), moreover, elicited feeding rejection at medium concentrations within richest assay foods. 

The most potent feeding repellents were by far the meridianins A–G, followed by the rossinone B, and then glassponsine; 5α(*H*)-cholestan-3-one and wax esters presented lower activities. According to what is routinely assumed about antipredatory chemistry, secondary metabolites (alkaloids **3**–**9**, meroterpene **10** and sulfonate acid **12**) demonstrated significantly higher levels of defense compared with compounds deriving from primary metabolic routes (wax esters **1**–**2** and steroid **11**). Moreover, the fact that meridianin fractions were found as the most potent feeding repellents at any concentration, agrees with traditional theories, in that nitrogen-containing metabolites (such as alkaloids) should be bioactive at much lower concentrations than non-nitrogenous defenses such as phenolics or terpenes [1]. Some inorganic components (e.g., spicles, sclerites, calcified plant structures) have been attributed a defensive role, as to be able to act in a synergistic way with repellent metabolites against predators [12]. This synergism was not intended to be analyzed here, as sand was added to the prepared foods only to maintain a constant dry weight while changing the energetic content. No enhanced bioactivities were observed either way with pearls containing sand compared with sand-free ones, in combination with repellents. Remarkably, calculations of natural concentrations here and in most published studies, consider portions of animals that might not be susceptible to a cellular or glandular specific allocation of defensive metabolites, being thus quite conservative. Therefore, at smaller scales, these compounds could be even more concentrated, providing stronger effects. This was not a main topic of the present study, but it is important to consider an antipredatory activity of products at higher concentrations than those calculated as natural. In fact, according to the optimal defense theory (ODT), an optimal anatomical storage of defenses is important also to avoid possible autotoxicity [3,8].

## 3. Experimental Section

### 3.1. Sample Collection and Extraction

Selected Antarctic invertebrate samples from the Eastern Weddell Sea, collected by trawling between 228 and 309 m depth during the ANT XXI/2 cruise (November 2003–January 2004) on board the R/V Polarstern (AWI—Alfred Wegener Institute, Bremerhaven, Germany), were recovered for the isolation of the target metabolites. These collections included colonial ascidian samples of the species *Aplidium falklandicum* Millar, 1960 and *Aplidium fuegiense* Cunningham, 1871; and hexactinellid sponge samples, *Anoxycalyx* (*Scolymastra*) *joubini* Topsent, 1916 and *Rossella nuda* Topsent, 1901. Moreover, colonies of the soft coral *Alcyonium haddoni* Wright and Studer, 1889 were collected at 15 m depth by scuba diving around Deception Island (South Shetland Archipelago, Antarctica) during the ACTIQUIM-2 campaign (December 2009–January 2010). Each single sample comprised several colonial clumps or individuals of each species from a collection site, which were grouped together for further experimentation and analysis. Pictures of fresh animals were taken on board, and a voucher portion of each sample was conserved in 10% formalin for taxonomy. The material was frozen at −20 °C, and sent to the University of Barcelona, where all five samples were classified to species level (Table 1).

Each of the five samples, consisting of various colonies/specimens, was thawed, cut in small pieces and extracted with acetone at room temperature while grinding the tissue with a mortar and pestle. In the particular case of *Aplidium fueginese* colonies were previously dissected into inner and outer body regions, and only the inner region (zooids), reported to store the target metabolite rossinone (**10**) [21,28], was processed. After removal of the solvent *in vacuo*, residual water was partitioned three times with diethyl ether (Et_2_O) and once with *n-*butanol. The organic phases were combined to obtain an ether fraction (EE) and a *n-*butanol fraction (BE). The respective organic solvents were evaporated under reduced pressure, providing dry EE and BE fractions and an aqueous residue. The dry crude fractions were weighed for calculations of yield. The weight of the material of the residual aqueous fraction was not included in the dry weight calculation. Four ether and one *n*-butanol fractions, each one obtained from one of the five samples of the analysis, were used for the chemical purifications of the five target metabolites **1**–**12** (see Section 3.3). Tissue concentrations for the isolated compounds, hereafter referred to as “natural concentrations,” were calculated with respect to a sample’s total dry weight: DW_TOTAL_ = DW dry weight of the solid extracted remains + EE dry weight + BE dry weight (Table 1). We chose dry weight for tissue concentration calculations because it avoids issues of high variability of weight parameters related to water content in aquatic, soft-bodied samples.

### 3.2. Molecular Characterization

^1^H and ^13^C NMR spectra of samples dissolved in CDCl_3_ were recorded on DRX 600, Avance 400, and DPX 300 MHz Bruker spectrometers (Bruker BioSpin AG, Fällanden, Switzerland), with chemical shifts reported in ppm relative to CHCl_3_ (δ 7.26 for proton and δ 77.0 for carbon). Electro Spray Ionization Mass Spectroscopy (ESIMS) and High Resolution Electron Spray Ionization Mass Spectroscopy (HRESIMS) were measured on a Micromass Q-TOF Micro mass spectrometer coupled with a Waters Alliance 2695 HPLC (Waters S.A.S., En Yvelines Cedex, France). The instrument was calibrated with a polyethylene glycol (PEG) mixture representing molecular weights ranging from 200 to 1000 g/mol. Silica gel chromatography was performed using precoated Merck F254 plates and Merck Kieselgel 60 powder (Merck Millipore, Vimodrone (MI), Italy). HPLC purification was carried out on a Shimadzu LC-10 AD liquid chromatograph equipped with a UV SPD-10A wave-length detector (Shimadzu Europa GmbH, Duisburg, Germany).

### 3.3. Purification of Target Metabolites

Crude organic extracts (diethyl ether and butanol) obtained from our five invertebrate samples were transferred to ICB-CNR (Pozzuoli, Napoli, Italy), where they were further processed. They were screened by TLC using light petroleum ether/Et_2_O in different ratios (1:0, 8:2, 1:1, 2:8, 0:1), and chloroform/methanol 8:2. The plates were developed with CeSO_4_ revealing diverse spots, depending on each particular sample. Organic extracts containing the selected metabolites of interest for the study were fractionated through chromatography on Sephadex LH-20 (Sigma-Aldrich, Milano, Italy) with a 1:1 mixture of chloroform/methanol, and/or were submitted to silica gel (Merk Kiesegel 60, 0.063–0.2, Merck Millipore, Vimodrone (MI), Italy) column purification using light petroleum ether/Et_2_O/chloroform/methanol as eluents in different combinations and ratios, according to the nature of the target compounds. The following five metabolites were used for the bioassays (see Table 1):

- **Wax esters (1–2):** The lipophilic (Et_2_O) fraction from the soft coral *Alcyonium haddoni* was submitted to silica gel column purification to give a wax ester subfraction (*Rf* 0.9, light petroleum ether/Et_2_O, 9:1). LC-MS analysis (250 × 4.60 mm, Phenomenex Kromasil C18, 60 min gradient from 30% to 100% CH_3_OH in H_2_O) showed that the wax ester mixture comprised the two main components: wax (**1**) C34:1 (*m/z* 529 [M + Na]^+^), and wax (**2**) C32:1 (*m/z* 501 [M + Na]^+^) (both composed of a C18:1 monounsaturated fatty acid, and C16:0 and C14:0 saturated alcohol esters, respectively). **1**–**2** were assayed as a mixture, because they are the main components of the wax ester fraction in *Alcyonium* soft corals. For further details on purification and molecular determination of products **1** and **2** see [27];
- **Meridianins A–G (3–9):** Column chromatography of the ether fraction of the colonial ascidian *Aplidium falklandicum*, followed by a TLC purification with preparative (SiO_2_) plates (Merk Kiesegel 60 F254 0.50 and 1.00 mm), provided an abundant yellowish subfraction (*Rf* 0.63, chloroform/methanol, 8:2), which corresponded to the alkaloid mixture of meridianins A–G. These products (**3**–**9**) were tested as a mixture since in nature they occur together as a rich fraction of bioactively related derivatives. For details on purification and molecular determination of meridianins A–G see [21,25];
- **Rossinone B (10):** The Et_2_O inner extract from the ascidian *Aplidium fuegiense* was fractioned through column and TLC purifications, allowing to recover significant quantities of the meroterpenoid rossinone B (**10**) (*Rf* 0.65, light petroleum ether/Et_2_O, 2:8). Details on purification and molecular determination of product **10** may be found in [21,28];
- **5α(*H*)-cholestan-3-one (11):** The ether extract of the glass sponge *Rossella nuda* was fractioned by silica gel chromatography, using a gradient of light petroleum ether/Et_2_O. The fraction eluted with 10% of diethyl ether (*Rf* 0.51, light petroleum ether/Et_2_O, 8:2) contained pure 5α(*H*)-cholestan-3-one (**11**). More information on the isolation of product **11** is published in [26];
- **Glassponsine (12):** The fractionation of the BE fraction of the hexactinellid *Anoxycalyx (Scolimastra) joubini* on Si gel column chromatography (gradient 0%–100% metanol in chloroform) yielded a fraction containing a UV sensitive spot at *Rf* 0.15 (chloroform/methanol, 8:2). This fraction was further purified by preparative TLC chromatography (SiO_2_, chloroform/methanol, 65:35) to yield a recently reported pure compound, glassponsine **12**, with a structure elucidated by spectroscopic methods elsewhere [29].

### 3.4. Artificial Food Preparation

Alginate caviar-textured feeding pearls were used in the assays. Gelling agents add little nutritional value, and the resulting artificially prepared diets allow homogeneous inclusion of ingredients, while controlling the nutritional content (determined by the quantity of food supplement added), and the concentration of metabolites under investigation. An added advantage of using alginate is that, unlike other gelling agents, it does not require heating, reducing chemical degradation. Phytoplan^®^, a spray-dried blend of several strains of phytoplankton (used for aquarium filter feeding invertebrates) was in our case the feeding source (30.3% protein, 13.6% carbohydrate and 13% of lipids, based on nutrition facts). Invertebrate tissues contain nutritionally valuable (e.g., protein) and inert components (e.g., mineralized skeletal inclusions: glass and/or calcareous spicules of sponges, and calcitic sclerites of corals and ascidians). Assay foods should include an equivalent mass of nutritionally inert matrix to mimic the mineralized inorganic skeletal elements naturally present in the groups studied. This was substituted by fine washed beach sand (0.0625 mm particule-size). Artificial diets were made with Kit Sferificacion^®^ [49] similarly as in previous studies by our group [19,21,26,27]. Food pearls were prepared by mixing 5 mg·mL^−1^ of alginate (Algin^®^ of Kit Sferificacion^®^) aqueous solution, along with three different quantities/concentrations of Phytoplan^®^ (200, 100 and 50 mg; 66.7, 33.3 and 17 mg·mL^−1^), a compensating quantity of sand (0%, 50% and 75% of food’s total dry mass) to maintain constant dry weight in the prepared artificial food mixes, and a drop of green or red food dye (Table 4; see next paragraph). This provided the production of three diets with constant dry weight (≈200 mg) but different nutritional quality, estimated in energetic values as ≈19, 9.5 and 4.75 KJ·g^−1^ in dry weight, respectively (according to the Atwater factor system [50]). Published analyses from several authors report energetic values of 5–6 KJ·g^−1^ dry wt in Antarctic hexactinellid sponges, which have a high spicule content and low organic matter and represent poorly attractive prey items [51,52]; whereas Antarctic soft corals and ascidians are instead highly energetic, attractive prey accounting for 16 KJ·g^−1^ and 15 KJ·g^−1^ DW (dry weight), respectively [53,54]. Assay diets in the current study were designed in order to obtain ranges of nutritional content (4.75–19 KJ·g^−1^ DW) ecologically reasonable and realistic according to the literature (to the mentioned analyses). The five types of isolated metabolites tested: wax esters (**1**–**2**), meridianins A–G (**3**–**9**), rossinone B (**10**), 5α(*H*)-cholestan-3-one (**11**) and glassponsine (**12**) were incorporated separately in the artificial foods via Phytoplan^®^, each at three different quantities: 2, 1, and 0.5 mg (from now on expressed in concentration: 10, 5, 2.5 mg·g^−1^_TOTAL·DW_). Compounds were dissolved in appropriate solvent carrier (either diethyl ether or methanol) to totally wet the dry components (food stimulant + sand), and the solvent was then evaporated, resulting in a uniform coating of the compound on the powdered food concentrate and sand prior to being added to the alginate aqueous mix. The new metabolite, glassponsine (**12**), was moreover assayed at its natural tissue concentration in preliminary tests, (2.34 mg·g^−1^ [29]; Table 1). The three assay compound concentrations: [**Conc**] = 10, 5, 2.5 mg·g^−1^_TOTAL·DW_, were chosen based on 5 mg·g^−1^ being the active concentration in preliminary trials and previous studies [21,25,26,27]. Control food pearls were prepared identically but without including testing compounds, adding an equal volume of solvent alone. The diverse mixtures obtained were introduced into a syringe and added drop-wise to a 0.09 M (1%) CaCl_2_ (Calcic^®^ of Kit Sferificacion^®^) water solution, where they gelatinized (*i.e.*, polymerized) forming spheroid pearls of approximately 2.5 mm in diameter. The relative quantity of the ingredients required to produce a whole set of untreated control pearls for a single experiment was: 3 mL alginate solution + 200/100/50 mg Phytoplan^®^ + 0/100/150 mg sand respectively (200 mg dry components). This amount guarantees the formation of 150 pearls (15 replicates × 10 pearls per replicate) as well as extra pearls for pilot trials investigating feeding-unrelated changes to the pearls (see next subsection). For compound-treated pearls, the three diet types also included each of the compounds incorporated at the three different concentrations. Thus, each of the five compound types (**1**–**2**, **3**–**9**, **10**–**12**) at three different concentrations ([**Conc**]’s), was separately incorporated in the three diets of different nutritive content, resulting in nine assays per metabolite (3 [**Conc**]’s × 3 diets), and a total of 45 experiments for the whole study (9 assays × 5 compounds; see Table 4; Figure 1). Feeding experiments with small food pearls allows the measuring of “defense activity per pearl”. 

**Table 4:** Feeding matrix for all treatments and controls. Composition of the 45 treatment assay food pearls (containing compounds), and for the three types of control diets (compound-free) of the study, showing the relative quantities of the ingredients required to prepare a set of feeding pearls for a single experiment. [**Conc**] DW_TOTAL_ (mg·g^−1^): Compound concentration respect to the total dry weight of the artificially prepared diet; [**Conc**] Vol (mg·mL^−1^): Compound concentration respect to the volume of the feeding pearls. * A drop of food coloring (red or green) was added to all feeding alginate-pearl mixes at the end, before the spherification process.

Compounds Tested	Composition of Artificially Prepared Diets (Feeding Pearls) Compound (mg)/PhytoPlan (mg)/Sand (mg)/Alginate Solution (mL)			[Conc] DW_TOTAL_ (mg·g^−1^)	[Conc] Volume (mg·mL^−1^)
Wax esters mix (**1–2**) Meridianins A–G mix (**3–9**) Rossinone B (**10**) 5α(*H*)-cholestan-3-one (**11**) Glassponsine (**12**)	2/200/0/3	2/100/100/3	2/50/150/3	10	0.66
	1/200/0/3	1/100/100/3	1/50/150/3	5	0.33
	0.5/200/0/3	0.5/100/100/3	0.5/50/150/3	2.5	0.17
CONTROLS	0/200/0/3	0/100/100/3	0/50/150/3	-	-

Control and treatment pearls (containing isolated compounds) were visually distinguished in paired assays by adding different liquid tasteless food dyes (red and green) to the alginate mix before spherification in CaCl_2_ solution. Pilot trials confirmed that feeding preferences of *Cheirimedon femoratus* did not differ between colored and uncolored pearls (*p* = 0.47), or between red and green colored pearls (*p* = 0.47). Nevertheless, control and treatment food pearls were randomly swapped to green or red colorations throughout the experimentation period. Paired preference assays were also performed with control pearls of the three different diet types (200, 100 and 50 mg of Phytoplan^®^), in order to contrast the preferences and consumption rates of the amphipod towards more or less energy-rich diets.

### 3.5. Feeding-Preference Bioassays with Amphipods

Lyssianasid amphipods of the abundant, eurybathic Antarctic species *Cheirimedon femoratus* were chosen for feeding experiments for representing appropriate realistic generalist predators. The base protocol applied was described in [19], and it has been previously used in other studies testing isolated compounds [21,25,26,27]. These amphipods are voracious opportunistic feeders with a circumpolar distribution [55,56]. Thousands of individuals were captured by scuba diving in Port Foster Bay (Deception Island, South Shetland Archipelago: 62°59.369ʹ S, 60°33.424ʹ W) with fishing nets, between 2 and 7 m depth during the Antarctic cruise ACTIQUIM-3 (January 2011–March 2012). Once the experiments were completed, living specimens were returned to the sea. Amphipods were starved for 3 days while maintained in 8 L aquariums at the labs of the Spanish Base BAE “Gabriel de Castilla” (Deception Island) where the bioassays took place. Each assay consisted of 15 replicate containers filled with 500 mL of sea water and 15 amphipods, which were offered a simultaneous choice between 10 treatments (incorporating isolated compounds) and 10 control pearls (see Figure 1). When food items are small (<5 mm diameter) changes in mass due to consumption are negligible, and thus consumption or rejection is scored as the number of individual pellets [3]. The assays ended 5 h after food presentation, and the number of leftover pearls of each color (control or treatment) was then recorded. A food pearl was considered eaten when it was ingested up to remaining ~1/8 its original size (using a 1 mm^2^ mesh template). Since our feeding trials were short in time, mass autogenic alterations were avoided, and there was no need to run controls in the absence of amphipods for changes unrelated to consumption [57]. Uneaten or unused extra treatment pearls were assessed by TLC screenings to check for possible alterations related to chemical degradation or loss of the test compounds. TLC does not provide exact compound concentrations, but it may reveal a rough chemical profile of the metabolites present within a chemical extract/fraction, in situations when accessibility to more sophisticated equipment is limited (e.g., Antarctica). Since experimental timings were short (5 h), and Antarctic seawater temperature is very cold (≈1 °C), the modification/loss of organic products into the water column should be negligible.

### 3.6. Statistical Analysis

Differences in the quantity of ingested control and treated foods were calculated by counting the number of remaining uneaten pearls. Changes in the amount of the two foods in each container are not independent and have correlated errors. Each replicate is thus represented by a paired result yielding two sets of data (treatment and control). Since assumptions of normality and homogeneity of variances were not met, data were compared by non-parametric procedures applying Exact Wilcoxon Tests. The average ingestion rates for the three types of compound-free control diets holding different energetic content (made with 200, 100 and 50 mg of Phytoplan^®^) were calculated considering the data recorded for control feeding pearls (in 5 h exposure) in all the experiments performed of the study (45 assays in total). Data were evaluated for normality using Kolmogorov–Smirnov and Shapiro tests and for homogeneity of variance with Bartlett. Significant differences in the average ingestion rates in the three base control diets was contrasted by applying a one-way analysis of variance (ANOVA, α = 0.05), followed by a Tukey (HDS) *post hoc* test to determine two by two differences. The interaction between feeding deterrence and energetic content of assay foods was assessed statistically. Contingency tables 3 × 3 were constructed for each of the five compound types assessed (Wax esters **1**–**2**, Meridianins **3**–**9**, Rossinone **10**, 5α(*H*)-cholestan-3-one **11**, Glassponsine **12**), with the two variables “Assay diet” and “Compound concentration”. Assay diet had three categories, corresponding to the three base diets of different energetic content (pearls prepared with 200, 100 and 50 mg of Phytoplan^®^); while compound concentration included the three categories of product quantity (10, 5 and 2.5 mg·g^−1^ DW_TOTAL_) incorporated into the artificial foods. For each of the experiments, we calculated the average of the difference in consumption between paired control (compound-free) *vs.* treatment (with compound) feeding pearls within the 15 replicate tests, and further divided this value by the average ingestion rate of the 15 control pearl-based foods of that experiment. This value was then inserted in the corresponding contingency table as percentage: *i.e.*, **∂**_PhytoPlan:[**Conc**]_ (see Supplementary Figure S1). 


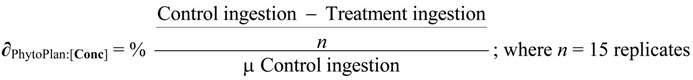


G-tests of independence with Williams’s correction were then applied to determine whether the two categorical variables are associated with one another (Diet energetic content *vs.* Compound concentration), in relation with the bioactivity of each of the compound types assayed in the distinct conditions. The larger the G value (likelihood ratio) is, the greater is the probability (and level) of interaction/dependence between the two variables (and the smaller the *p*-value). The null hypothesis in the G-test is that the relative proportions of one variable are independent of the second variable, and significant tests (*p* < 0.05) reveal interference between variables.

All tests and most graphing material were performed on R-command software using R Studio, version v0.98.507, run with R statistics package, version 3.1.0 [58].

## 4. Conclusions

Antarctic invertebrates, and in general marine organisms, extensively use chemical defenses to control predation. Nevertheless, much needs to be learned about how antipredatory agents operate [2,5,17,18]. With this study, we showed that there is no relationship between the level of potency of a defensive chemical and the concentration in which it appears in the organisms’ tissues. The most naturally abundant metabolites, meridianins and waxes, were respectively the most and the least active deterrents. Moreover, each compound may exhibit variable levels of repellency, and they may perform differently when mixed with distinct assay foods. The natural products that interacted the most with energetic content were those occurring in nature at higher concentrations, again meridianins and wax esters. The remaining metabolites (rossinone, 5α(*H*)-cholestan-3-one and glassponsine) require a threshold concentration to elicit feeding repellence, regardless of nutritional quality. The efficacy of a defensive product as repellent (in terms of minimal concentrations to afford activity), and the way it may interact with high energy foods is difficult to predict and seems to be characteristic of each compound type. Indeed, different types of compounds may interfere very uniquely with dietary components. Several authors have proposed that, even if it has not been sufficiently investigated, the way nutrients may mask the stimuli that elicit avoidance is by these components attaching to specific moieties of deterrent metabolites. Nutrient attractants might, in such a manner, compete for binding sites of taste receptors offsetting repellents’ effect. Such receptors seem to be, indeed, highly conserved in different taxa, in terms of biochemical constituents, ([7] and references therein). An exciting issue for future research would be to elucidate the interactions between nutrients and defensive metabolites at the molecular level. 

## Supplementary Files

Supplementary File 1 Supplementary Information (PDF, 282 KB)
