# Supplementary material for: Defensive Metabolites from Antarctic Invertebrates: Does Energetic Content Interfere with Feeding Repellence?
Source: Mar Drugs. 2014 Jun 24;12(6):3770–91. doi: 10.3390/md12063770 (PMC4071601; doi:10.3390/md12063770)
Supplement: Supplementary File 1 — Supplementary Information (PDF, 282 KB) [file marinedrugs-12-03770-s001.pdf]

Supplementary Information

Figure S1. Diagram showing the repellent bioactivities of the target metabolites in 45 feeding preference experiments performed with the amphipod *Cheirimedon femoratus*. Five types of compounds (**1**–**12**) were tested at three different concentrations, and were incorporated into three assay diets of distinct energetic values (5 **Comp** × 3 [**Conc**] × 3 diets = 45 bioassays). The three types of artificially prepared diets (feeding alginate pearls) contained 200, 100 and 50 mg of Phytoplan® feeding stimulant (and a compensating dry weight content of sand of: 0, 100 and 150 mg, respectively), and are represented along the X-axis with different shaded areas. On the Y-axis, assay testing concentrations of metabolites (**1**–**12**) are expressed respect to the total dry weight: 10, 5 and 2.5 mg g^{-1} DW_{TOTAL}. These values correspond to the quantities of compounds added in the treatment foods respectively (2, 1 and 0.5 mg **Comp**). The five compound types are symbolized with icons: "Wax"—circle: Wax esters (**1**–**2**); "Mer"—triangle: meridianins A–G (**3**–**9**); "Ross"—rombe: rossinone B (**10**); "Chol"—star: 5α(*H*)-cholestan-3-one (**11**) and "Glass"—moon: glassponsine (**12**). Only active results of feeding repellency for each metabolite in the bioassays, at a certain [**Conc**] and diet, are shown in the graphic with the corresponding compound icons. Repellent activities correspond to significant lower ingestion rates in treatment feeding pearls (compound-containing) respect to paired control pearls (compound-free), analyzed with Wilcoxon Exact Tests: Active in feeding repellence ($p < 0.05$ *); Inactive ($p \geq 0.05$ n.s.).

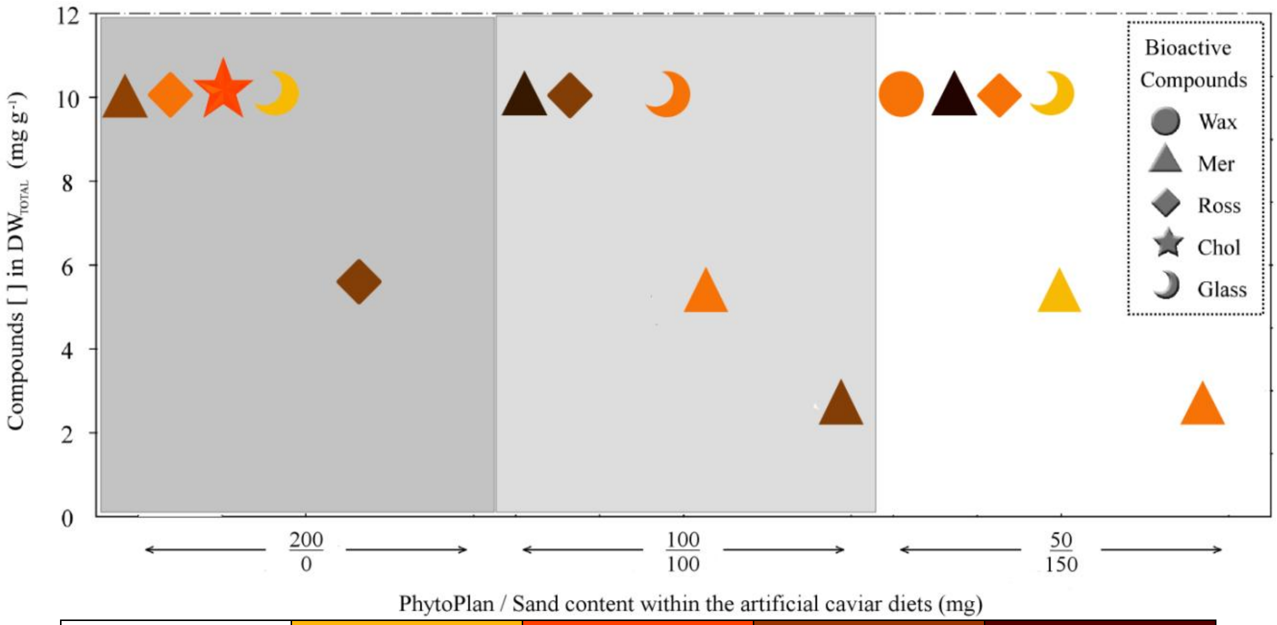

0%–20%	21%–40%	41%–60%	61%–80%	81%–100%

* Color codes corresponding to the percentage of the average difference of ingestion rate between Control *vs.* Treatment food pearls for the 15 replicate tests in each experiment.

Table S1. Example of contingency tables 3 × 3 constructed with the categorical variables Assay diet, "Diet", and Compound concentration, "[**Conc**]" were confronted, for each of the five compound types **1**–**12** assessed in the feeding preference experiments with *Cheirimedon femoratus* (Wax esters **1**–**2**, Meridianins **3**–**9**, Rossinone **10**, 5α(*H*)-cholestan-3-one **11**, Glassponsine **12**). Values in the table were calculated as: $\partial_{\text{PhytoPlan:[}\mathbf{Conc}\text{]}}$ = % (((Control ingestion − Treatment ingestion)/*n*)/µ Control ingestion); *n* = 15 replicate tests.

Diet / [Conc] *	200 mg Phyto	100 mg Phyto	50 mg Phyto
10 mg g^{-1}	$\partial_{10:200}$	$\partial_{10:100}$	$\partial_{10:50}$
5 mg g^{-1}	$\partial_{5:200}$	$\partial_{5:100}$	$\partial_{5:50}$
2.5 mg g^{-1}	$\partial_{2.5:200}$	$\partial_{2.5:100}$	$\partial_{2.5:50}$

* **Comp**: Wax esters **1**–**2**; Meridianins **3**–**9**; Rossinone **10**; Cholestan**11**; Glassponsine **12**.

$$\partial_{\text{PhytoPlan:[}\mathbf{Conc}\text{]}} = \% \, \frac{\frac{\text{Control ingestion} - \text{Treatment ingestion}}{n}}{\mu \text{ Control ingestion}}\text{; where } n = 15 \text{ replicates}$$

© 2014 by the authors; licensee MDPI, Basel, Switzerland. This article is an open access article distributed under the terms and conditions of the Creative Commons Attribution license (http://creativecommons.org/licenses/by/3.0/).
